# Development and evaluation of a bovine lung-on-chip (bLOC) to study bovine respiratory diseases

**DOI:** 10.1007/s44164-022-00030-z

**Published:** 2022-08-17

**Authors:** Diane F. Lee, Clare L. Thompson, Ronald E. Baynes, Hiroko Enomoto, Geof W. Smith, Mark A. Chambers

**Affiliations:** 1 School of Veterinary Medicine, University of Surrey, Guildford, UK; 2 College of Veterinary Medicine, North Carolina State University, Raleigh, NC USA; 3 Zoetis Inc., Raleigh, NC USA; 4 Now at Sussex Drug Discovery Centre, University of Sussex, Falmer, UK; 5 Centre for Predictive In Vitro Models, School of Engineering and Materials Science, Queen Mary University of London, London, UK

**Keywords:** Bovine respiratory disease, Organ-on-chip, Lung, In vitro, Pharmacokinetics, 3Rs

## Abstract

**Purpose:**

Current air-liquid interface (ALI) models of bovine proximal airways have their limitations. They do not simulate blood flow necessary to mimic systemic drug administration, and repeated sampling requires multiple, independent cultures. A bovine lung-on-chip (bLOC) would overcome these limitations, providing a convenient and cost-effective model for pharmacokinetic or pathogenicity studies.

**Methods:**

Bovine pulmonary arterial endothelial cells seeded into the endothelial channel of an Emulate Lung-Chip were interfaced with bovine bronchial epithelial cells in the epithelial channel. Cells were cultured at ALI for up to 21 days. Differentiation was assessed by mucin quantification, phase-contrast light microscopy and immunofluorescence of cell-specific markers in fixed cultures. Barrier integrity was determined by FITC-labelled dextran 3–5 kDa permeability. To evaluate the model, endothelial-epithelial transport of the antibiotic drug, danofloxacin, was followed using liquid chromatography-mass spectrometry, with the aim of replicating data previously determined in vivo.

**Results:**

bLOC cultures secreted quantifiable mucins, whilst cilia formation was evident in the epithelial channel. Barrier integrity of the model was demonstrated by resistance to FITC-Dextran 3–5 kDa permeation. Bronchial epithelial and endothelial cell-specific markers were observed. Close to plasma, representative PK data for danofloxacin was observed in the endothelial channel; however, danofloxacin in the epithelial channel was mostly below the limit of quantification.

**Conclusion:**

A co-culture model of the bovine proximal airway was successfully generated, with potential to replace in vivo experimentation. With further optimisation and characterisation, the bLOC may be suitable to perform drug pharmacokinetic studies for bovine respiratory disease (BRD), and other applications.

## Introduction

Calf pneumonia, or bovine respiratory disease (BRD) complex, is a major economic threat to the cattle industry [1]. The disease complex is caused by a variety of pathogens: viral, bacterial, parasitic, and fungal. Most typically, the clinical disease is caused by *Pasteurella multocida*, *Mannheimia haemolytica* or *Histophilus somni* bacteria that enter the lower respiratory tract after the airways have been compromised by primary infection with either *Mycoplasma bovis* or a virus (typically bovine respiratory syncytial virus (BRSV), parainfluenza 3 (PI3), bovine adenovirus-3 or 7 (BAV-3/7), bovine viral diarrhoea virus (BVDV), bovine herpes virus-1 (BHV-1)). Environmental conditions and/or other stress factors, such as weaning, changes of feed, variation in ambient temperature and humidity, also play an important part in the susceptibility of the calf to developing pneumonia [2]. Recent advances in antimicrobials and new vaccines have failed to remove BRD, stressing the importance of closing the gaps in our knowledge of how BRD pathogens interact with and evade host immunity. One early line of defence lies with the airway epithelial cells, which act in concert to remove pathogens via the muco-ciliary clearance (MCC) system or induce downstream innate immune responses via such mechanisms as pattern recognition receptor (PRR) signalling and the secretion of antimicrobial peptides. Ciliated cells are particularly susceptible to viral pathogenic disruption, being subjected to ciliary dysfunction and necrosis and rendering the MCC ineffective, thus paving the way for opportunistic, secondary, bacterial infection [3, 4].

Whilst in vivo studies have helped elucidate the processes involved in BRD, it is difficult to follow the transient, early events of host-pathogen interactions in the whole organism. Static air-liquid interface (ALI) models currently exist for short-term culture of bovine ciliated airway epithelia, including a monoculture of bovine bronchial epithelial cells (BBECs) [5] that was recently used to study invasion of airway epithelial cells by *M. haemolytica* [6]; however, more sophisticated and dynamic ‘lung-on-chip’ models are currently missing from the bovine research portfolio. The development of advanced models is driven by a need to streamline the drug discovery process (particularly with regard to increasing throughput and facilitating the acquisition of pharmacokinetics data), fill in knowledge gaps with regard to pathogen and drug interaction with the respiratory surface mucosa and a wish for the more ethical use of animals in basic research and drug development (the 3R principles of Replace, Reduce, and Refine) [7, 8]. Existing bovine models have limitations for the determination of pharmacokinetics (PK) parameters of drugs, or for the prediction of toxicity of novel therapeutics. First, static models do not recreate the flow of blood necessary to mimic systemic drug administration and delivery of drugs to the lung. Second, repeated sampling of the airway surface is problematic, and multiple independent cultures are required to generate experimental replicates, with no guarantee of reproducibility. These limitations underlie the importance of physiological relevance in such models. Species-specific in vitro models enable successful toxicological evaluation of novel therapies in addition to bioavailability, absorption and adsorption studies [9], but their findings are less meaningful if the replication of in vivo parameters has not been achieved, including representative barrier function and differentiation. More sophisticated models, such as those incorporating multiple cell types which constitute the organ of interest, could add credibility to the data generated. To date, such a species-specific, dynamic model does not exist for the bovine proximal airway. In other species, a number of perfused lung-on-chip (LOC) models make full use of micro-engineering advances, for example the introduction of ‘breathing-induced’ mechanical forces by way of a cyclic vacuum [10] onto porous membranes which effectively compartmentalise a microfluidic device. Combined with macro-porous scaffolds, formulated using extracellular matrix components such as collagen and elastin [11] or porous hydrogels [12], such LOC models are better able to mimic the shear stresses inflicted by cyclic strain. These ‘second generation’ models form a platform with which to investigate physiological or pathological effects and infection dynamics, including but not exclusive to the impact of physiological components such as surfactant on early bacterial or viral infection [13, 14]. Moreover, the flexibility of the cell scaffolds used has highlighted the effect of stretch on permeability, release of reactive oxygen species, cytokines and surfactant [15].

The bovine lung-on-a-chip (bLOC) described here is a novel and species-specific LOC tool which utilises the Emulate, Inc Chip S1®, a microfluidic device with two microchannels separated by a collagen I–coated porous (7 μm) membrane. This device has been previously used to assess the inhibitory activities of clinically approved drugs used to prevent infection of SARS-CoV-2 [14] and shows great promise in its potential to reduce reliance on in vivo experiments to evaluate drug PK. In the bLOC model, BBECs are seeded into the apical or ‘epithelial’ channel and are interfaced with bovine pulmonary arterial endothelial cells (BPAECs) on the underside of the membrane, in the basal or ‘endothelial’ channel. This study aims to assemble and characterise the bLOC model as proof-of-concept by assessing the model’s ability to replicate in vivo findings. To achieve this, pulse-chase experiments were performed to follow the endothelial to epithelial drug transport of danofloxacin, a commonly administered antibiotic previously demonstrated to be effective in the treatment of *P. multocida*–associated BRD in vivo [16]. Although we were unable to demonstrate in vitro/in vivo correlation (IVIVC) for danofloxacin, the data generated demonstrate the potential of bLOC for the study of drug transport, infection and PK studies in vitro, providing a more accurate assessment at the ALI, once the model system has been suitably modified and further characterised.

## Materials and methods

### Isolation of BBECs

Tissue was excised from the tracheal bifurcation of a cow aged <24 months, slaughtered for consumption by a local abattoir. The tissue was transported in Dulbecco’s phosphate-buffered saline (DPBS) (Thermo Fisher Scientific, Waltham, MA) and Primocin (100 μg/mL) (Invivogen, San Diego, CA) to the laboratory. The respiratory mucosal surface (total of ~40 mL by volume) was washed with fresh DPBS/Primocin before stripping mucosa from the submucosa using sterile forceps and a scalpel. These were dissected into pieces approximately 3 mm^2^ in size, which were washed four times in DPBS/Primocin by vigorous shaking. The pieces were then added to 50-mL tubes for enzymatic digestion, splitting to ensure that pieces did not occupy greater than 10 mL in volume per tube. Digestion solution (30 mL, consisting of trypsin 0.25 %/ethylene diamine tetra acetic acid sodium salt (EDTA) 1 mM (Thermo Fisher Scientific)/Primocin 100 μg/mL) was added to each tube, before incubation on a roller at 37°C for a total of 4 h. The pieces were then strained from each tube by serial filtration through cell strainers (Corning Inc., New York, NY) of 100 μm, 70 μm and 40 μm mesh sizing. Strained cells in suspension were spun at 300 × *g*, washed in DPBS/Primocin 100 μg/mL twice and resuspended in complete PneumaCult Ex (prepared to manufacturer’s recommendations) (PC-EX) (Stemcell Technologies, Vancouver, Canada), containing Primocin 100 μg/mL for counting on the BioRAD TC20 cell counter. Cells were seeded onto collagen I (ready to use solution, Thermo Fisher Scientific)–coated plastic at a density of 1 × 10^5^ cells/cm^2^. Cells were routinely passaged at 80 % confluence by a 1 in 2 split, by rinsing with DPBS and incubating for 5 min with 0.25 % trypsin/1 mM EDTA. Frozen stocks were prepared by pelleting 1st to 3rd passage cells (1 × 10^6^ per cryovial) and resuspending in 1 mL Cell Recovery solution (Corning Inc., Corning, NY) for storage in liquid nitrogen.

Primary bovine pulmonary arterial endothelial cells (BPAECs) were procured via the European Collection of Authenticated Cell Cultures (European Collection of Authenticated Cell Cultures, Salisbury, UK). These were revived from received frozen stocks and cultured to 80 % confluence in endothelial growth medium 2 (EGM-2) (PromoCell GmbH, Heidelberg, Germany). Cells were used up to four passages, passaging as per BBECs (above), passaging by no more than a 1 in 4 split.

### Organ chip culture

**Fig. 1:**
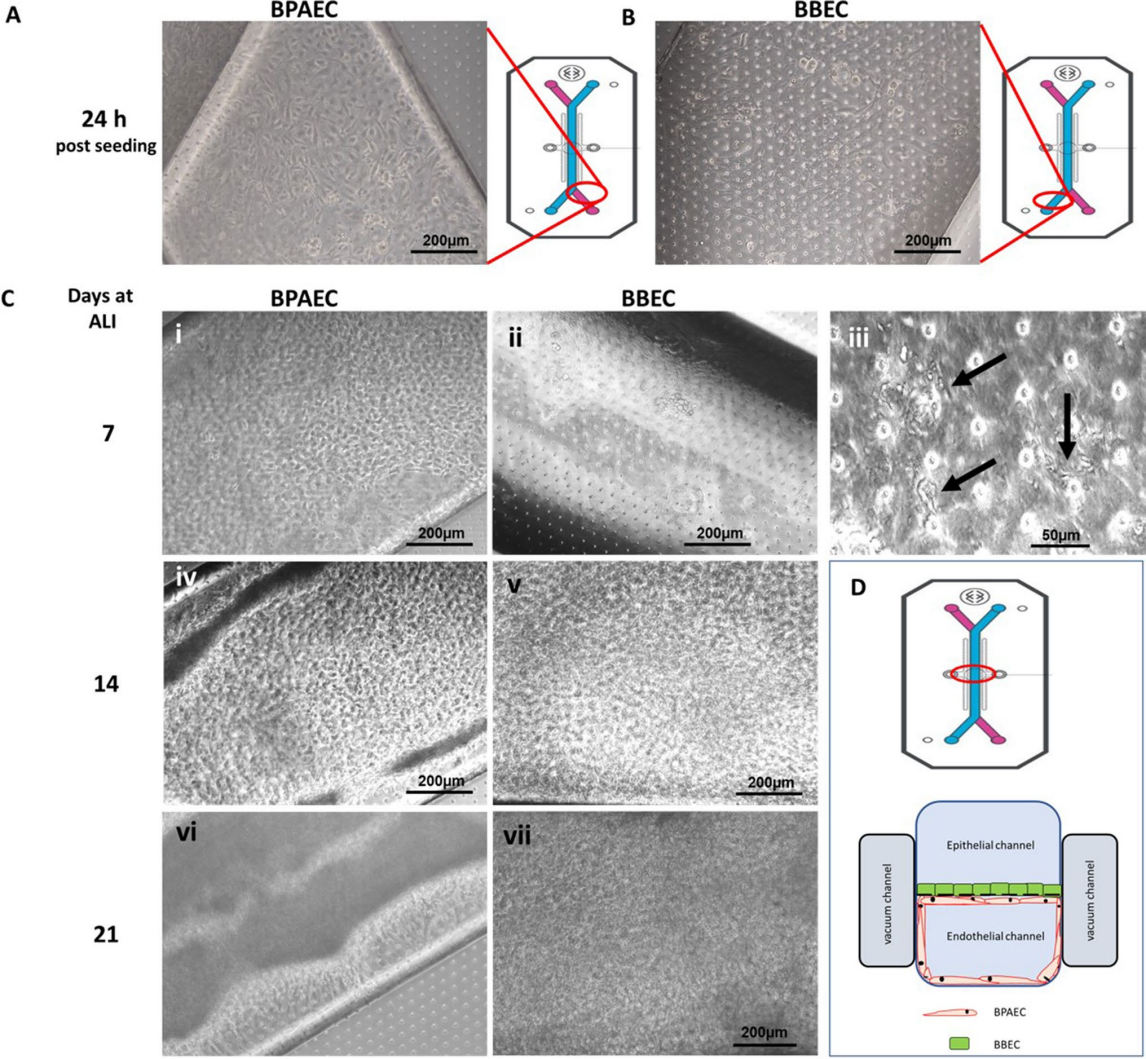
BPAECs were seeded first into the endothelial channel of the Emulate organ chip system (**A**), interfaced in the epithelial channel with BBECs (**B**), the two cell types separated by a porous (7 μm) membrane. Clear images of each cell type were acquired using the outlet ports (red circles) of the endothelial (pink) and epithelial (blue) channels. At 7 days, ALI, BPAEC (**C**i) and BBEC (**C**ii) cultures were already confluent, with clearly observed cilia in the epithelial channel (**C**iii, arrows). Both BPAEC (**C**iv) and BBEC (**C**v) were overconfluent at day 14, with multiple layers of BPAEC and excessive mucus in BBEC observed at day 21 (**C**vi and **C**vii respectively). A schematical representation of a transverse cross section of the chip shows the coverage of the endothelial channel by BPAEC and membrane culture of BBEC (**D**)

A bovine lung-on-chip (bLOC) was generated using the microfluidic 2-channel organ chip system (Emulate Inc., Boston, MA) (Fig. 1), comprising two adjacent parallel microchannels separated by a porous (7 μm) membrane. The poly-dimethylsiloxane (PDMS) surfaces of each channel were activated with 0.5 mg/mL ER-1 solution (Emulate Inc.), placing under a UV lamp for 10 min to activate the surface. This process was repeated before washing sequentially with ER-2 solution (Emulate Inc.) and DPBS. The membrane was then coated on both sides with collagen 1 from rat tail (50 μg/mL, CELL Applications Inc., San Diego, CA) at 4 °C overnight. Chips were flushed with EGM-2 prior to use.

BPAECs at 4th passage were seeded into the endothelial channel of organ chips at a concentration of 8 × 10^6^ cells/mL. Chips were inverted to encourage attachment to the underside of the membrane and incubated at 37°C for 2 h. Chips were then placed right side up and the epithelial (upper) channel washed with PC-EX medium. BBECs, at 1st to 3rd passage, were seeded to the epithelial channel at 1 × 10^6^ cells/mL and allowed to attach for a further 2 h at 37 °C. Both channels were then washed with their respective medium, and chips were cultured for a further 24 h at 37 °C with 5 % CO_2_ prior to connection to the ZÖE automated culture module (Emulate Inc.). Channels were perfused at a continuous volumetric flow rate of 30 μL/h. After 5–7 days, PC-EX medium was removed from the epithelial channel to establish an air-liquid interface (ALI) and the chip cultured for a further 14–21 days at 37 °C, 5 % CO_2_, fed via the endothelial channel with EGM-2. The epithelial channel was rinsed with DPBS twice weekly to remove cellular debris and mucus. No vacuum was applied to the chips in this experiment.

### Mucin detection by lectin assay

DPBS (300 μL) was flushed through the epithelial channel increasing flow rate to 1000 μL/h for 2 min. Chips were then incubated with DPBS for 1 h before DPBS was flushed through the epithelial channel for 2 min at 1000 μL/h. A sample of the eluent (100 μL) was added to a well of a 96-well clear bottomed, white walled assay plate in duplicate, alongside serial dilutions of porcine gastric mucin (Merck & Co., Inc., Kenilworth, NJ) to generate a standard curve of 50, 25, 12.5, 6.25, 3.125, 1.56, 0.78 and 0.39 ng/well. Mucin was allowed to bind overnight at 4°C, then each well was washed three times with DPBS/1.0 % gelatin/0.05 % Tween 20 (Wash Buffer, WB) (all Merck). The plate was blocked with 150 μL PBS/1.0 % gelatin for 1 h at 37°C, and then washed three times with 200 μL WB. Lectin from *Triticum vulgaris* conjugated to fluorescein isothiocyanate (FITC, 100 μL, 5.0 μg/mL) was added to all wells, and the plate returned to 37°C for 1 h. All wells were washed as before, then the plate was read on a ClarioStar Plus plate reader (BMG Labtech, Ortenberg, Germany) with an excitation wavelength 485 nm and emission wavelength 520 nm. Unknowns were intrapolated from the standard curve using GraphPad Prism v9.0.0 (GraphPad Software Inc., San Diego, CA).

### Immunofluorescence microscopy

Chips were manually rinsed with pre-warmed DPBS, fixed with 4 % paraformaldehyde (PFA) (Merck) for 30 min and washed again with DPBS before permeabilisation in immunofluorescence wash buffer (IF buffer [17]) containing 0.1 % Triton X-100 for 15 min. Chips were blocked for 1 h at room temperature in DPBS/5 % normal goat serum/0.1 % Triton X-100 (Merck). Primary antibodies, diluted 1/100 in blocking buffer, were applied to cells and incubated overnight at 4°C. Chips were washed three times with IF buffer and secondary antibody (diluted 1/200 in blocking buffer) applied for 1 h at room temperature in the dark. Cells were again rinsed three times with IF buffer before staining nuclei with NucBlue DNA stain (2 drops per mL) (Thermo Fisher Scientific) in IF buffer. This was removed and replaced with DPBS for imaging on a Zeiss LSM 710 confocal microscope, processing images using ImageJ [18]. Antibodies were as follows: EpCAM (orb10618, Biorbyt, Cambridge, UK), cluster of differentiation 31 (CD31) Clone HEC7 (MA3100, Thermo Fisher Scientific), ZO-1 (INV 40-2200, Thermo Fisher Scientific), β-catenin (8480T, Cell Signalling Technologies, Danvers, MA). Secondary antibodies were goat anti-mouse FITC-conjugated and goat anti-rabbit Texas red, both from Thermo Fisher Scientific.

### Reverse transcription polymerase chain reaction of cell-specific markers

For relative quantification of cell-specific markers, messenger RNA (mRNA) was harvested from chips at 7, 14 and 21 days (cultured at ALI), using the RNeasy RNA extraction kit (Qiagen, Hilden, Germany), resuspending in 30 μL RNAse free water. Total RNA was quantified using a Biodrop μLITE spectrophotometer and 500 ng used to generate complementary DNA (cDNA) in a reverse transcriptase reaction using the qScript cDNA synthesis kit (Quanta Bio, Beverly, MA), according to the manufacturer’s protocol, on a Techne 3Prime Personal Thermal Cycler (Cole Parmer Instrument Company, London, UK). Polymerase chain reaction (PCR) was performed on approximately 60 ng (by RNA quantification) of each sample, using Brilliant III™ Ultra-Fast Mastermix (Agilent Technologies, Santa Clara, CA) according to the manufacturer’s protocol and the following TaqMan™ Gene Expression Assays: *MUC5AC* accession number Hs01365616_m1 (cross-reactive to bovine) fluorescein amidite (FAM) labelled, *FOXJ1* accession number Bt04308989_m1 FAM labelled, *VWF* accession number Bt04317985_m1 FAM labelled, *CD34* accession number Bt03212327_m1 FAM labelled and GAPDH (housekeeper) accession number Bt03210911_g1 2′-chloro-7′phenyl-1,4-dichloro-6-carboxy-fluorescein (VIC) labelled (all Thermo Fisher Scientific), as duplex reactions and in triplicate, using a CFX96 Touch Real-Time PCR Detection System (BioRad Laboratories, Inc., Hercules, CA). Fold change in expression was calculated using the Livak method (2^−∆∆Ct^) [19], normalising to bovine total lung RNA (Thermo Fisher Scientific).

### Apparent permeability assay

Chips were temporarily returned to liquid-liquid interface (LLI) and medium containing FITC-dextran (3–5 kDa; 0.1 mg/mL) (Merck) was added to the endothelial channel. Organ chips were perfused for 2 h at 120 μL/h and the fluorescence intensity of the medium of the top and bottom channels measured at 520 nm (FLUOstar Optima spectrophotometer). The apparent permeability (*P*_app_) was calculated as per Eq. 1:

1 $${P}_{\mathrm{app}}=-\frac{Q_{\mathrm{R}}\ast {Q}_{\mathrm{D}}}{SA\ast \left(\ {Q}_{\mathrm{R}}+{Q}_{\mathrm{D}}\right)}\ast \ln \left[1-\frac{C_{\mathrm{R},0}\ast \left({Q}_{\mathrm{R}}+{Q}_{\mathrm{D}}\right)}{\left({Q}_{\mathrm{R}}\ast {C}_{\mathrm{R},0}+{Q}_{\mathrm{D}}\ast {C}_{\mathrm{D},0}\right)}\ \right]$$

where *P*_app_ is the apparent permeability in units of cm/s, *SA* is the surface area of the co-culture channel (0.17cm^2^), *Q*_R_ and *Q*_D_ are the flow rates in the receiving and dosing channels, respectively, in units of cm^3^/s, and *C*_R,0_ and *C*_D,0_ are the recovered concentrations in the receiving and dosing channels, respectively, in any consistent units.

### Pharmacokinetics of danofloxacin

**Fig. 2:**
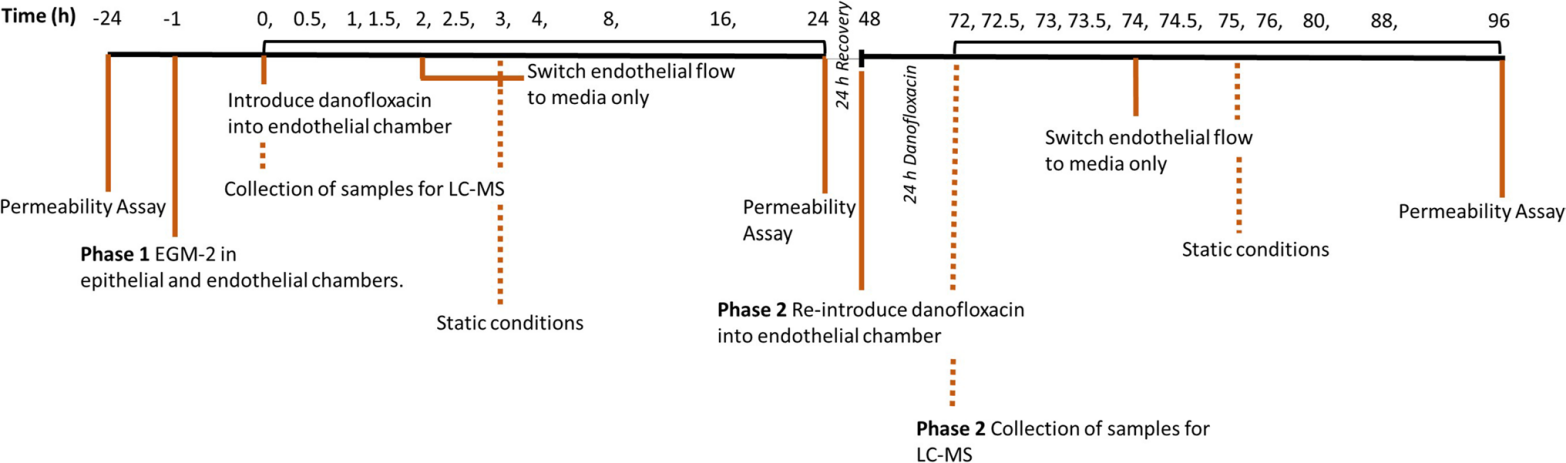
Schematic depicting the study of danofloxacin endothelial to epithelial drug transport in a two-phase ‘pulse-chase experiment’. Following a permeability assay and equilibration of the model, danofloxacin is introduced at 4 μg/mL under flow for 2 h in phase 1 and for a total of 26 h in phase 2. Samples were collected as indicated in the timeline

For PK analysis, chips were treated through the basal endothelial channel over two doses described as phase 1 and phase 2 (Fig. 2).

Phase 1: Danofloxacin (4 μg/mL) was prepared in EGM-2 and added to the endothelial channel (time point, 0 h) as a perfusion with volumetric flow rate of 300 μL/h for 2 h, fractionating the effluent from epithelial (top) and endothelial (bottom) channels every 30 min. After 2 h, the medium was replaced with fresh EGM-2 without danofloxacin, and the chips perfused for a further 1 h after which time chips were incubated under static conditions. Chip effluent was collected at 4, 8, 16 and 24 h, with flow reintroduced 30 min prior to each collection at 300 μL/h. At 24 h, chips were cultured under flow at 30 μL/h for a further 24 h (‘24 h recovery’) in the absence of danofloxacin, in preparation for phase 2.

Phase 2: Danofloxacin (4 μg/mL) was prepared in EGM-2 and added to the basal endothelial channel of the organ chip system (time point, 48 h). Media containing danofloxacin was perfused through the basal channel at a volumetric flow rate of 30 μL/h for 24 h. After 24 h (time point, 72 h), flow was increased to 300 μL/h for sample collection, from epithelial and endothelial channels every 30 min. At time point 74 h, the medium was again replaced with fresh EGM-2 only and the chips perfused for a further 1 h, following which chips were incubated under static conditions. Chip effluent was collected from the system at 4, 8, 16 and 24 h, with flow reintroduced 30 min prior to collection at 300 μL/h to flush the channel. All samples were snap-frozen and stored at −80 for ultra-performance liquid chromatography coupled with mass spectroscopy (UPLC-MS) analysis.

### UPLC-MS preparation of samples

All reagents were of analytical, high-performance liquid chromatography (HPLC) or LC-MS grade. Acetonitrile (ACN), acetic acid, methanol (MeOH), 29 % ammonium hydroxide and formic acid were supplied by Thermo Fisher Scientific. Trifluoroacetic acid (TFA), phosphoric acid, danofloxacin reference standard and sarafloxacin hydrochloride reference standard were purchased from Merck. Ultrapure water was supplied by Waters Corporation (Milford, MA).

Each sample (50 μL) was spiked with 5 μL of 10 μg/mL sarafloxacin, before adding 150 μL of 100 % ACN. The sample was subjected to vortex and centrifugation at 10,000 × *g* for 10 min. From this, 150 μL of supernatant was transferred into borosilicate glass tubes (VWR International, Radnor, PA). Phosphoric acid (150 μL of 4 %) was added and the solution mixed by vortex. Danofloxacin was extracted using an Oasis® MCX μElution plate 30 μm (Waters Corporation), preconditioned with 200 μL of 100 % MeOH followed by 200 μL of ultrapure water. The total 300 μL extracts were then added to the plate and passed through the plate under vacuum at ~ 3 psi. The plate was washed by 200 μL of 0.2 % formic acid prepared in water followed by 200 μL of 100 % MeOH under vacuum. The analyte was eluted into a 700-μL round 96-well sample plate (Waters Corporation) via 50 μL of 5 % ammonium hydroxide prepared in MeOH. Fifty microlitres of ultrapure water was added into eluents and mixed thoroughly.

Standards were prepared using EGM-2 in the range 0.05–10 μg/mL, using sarafloxacin as internal standard. Quality controls (0.15, 0.3, 3 and 7 μg/mL of danofloxacin) and blank (zero) media were injected with every batch.

### UPLC-MS conditions

UPLC-MS analysis of danofloxacin was performed using the Acquity Binary Solvent Manager (Waters Corporation), Sample Manager (Waters Corporation) and QDa Detector (Waters Corporation). Chromatographic separation was achieved by gradient elution on an Acquity UPLC® BEH C18 1.7 μm column (2.1 × 50mm) with Vanguard pre-column (Waters Corporation) at 35°C. The danofloxacin was detected in a single quad mass spectrometer operated with positive electrospray ionisation (ESI (+)) in selected ion recording (SIR) mode. The cone voltage was 20 V and capillary voltage was 0.6 V. The mobile phase solvents were 0.1 % TFA in water (A) and 100 % MeOH (B) at a flow rate 0.4 mL/min for 5 min. The gradient program started with 85 % of A 15 % of B, then 15 % of A 85 % of B (2.5–3.5 min) and immediately back to 85 % of A 15 % of B (3.51–5 min). The sample manager was maintained at 4°C and injection volume was 5 μL. Mass (m/z) of danofloxacin (M+H^+^) and sarafloxacin (M+H^+^) was 358 and 386, respectively. The retention time for danofloxacin and sarafloxacin was 2.10 and 2.19 min, respectively. The limit of detection (LOD) and the limit of quantification (LOQ) of danofloxacin were 0.05 and 0.1 μg/mL, respectively. The intraday precision and accuracy are shown in Table 1.

**Table 1:** Intraday precision and accuracy (*n*=6 replicates within 1 day) for EGM-2 samples. Six samples were spiked with danofloxacin at concentrations of 0.15, 0.3, 1.5, 3 and 7 μg/mL to validate the analytical assay for standard curve (0.05–10 μg/mL)

Analyte	Spiking concentration	Average estimate concentration	SD	CV	Recovery
				(%)	(%)
	(μg/mL)	(μg/mL)			
Danofloxacin	0.15	0.16	0.007	4.44	108.9
	0.3	0.29	0.008	2.75	96.2
	1.5	1.45	0.039	2.67	96.3
	3.0	2.82	0.144	5.11	94.0
	7.0	6.59	0.328	4.97	94.2

*SD*, standard deviation; *CV*, coefficient of variation

### Protein binding assay

Spiked EGM-2 (500 μL) at 0.3, 3 and 7 μg/mL danofloxacin (5 replicates) was allowed to equilibrate by standing at a room temperature for 30 min, then mixed thoroughly by pipetting. Supernatant (50 μL) was removed, and the sample preparation step performed as described previously to measure total concentration (protein bound danofloxacin + protein unbound danofloxacin). The remaining 450 μL was centrifuged by using Centrifree® ultrafiltration devices (MilliporeSigma, Burlington, MA) at 2000 × *g* for 20 min (Sorvall ST 16R centrifuge fixed angle bucket rotor, Thermo Fisher Scientific), to collect only protein unbound danofloxacin [20]. A sample (50 μL) was removed from the reservoir and processed by using the same method used to extract danofloxacin from EGM-2. EGM-2 only was centrifuged by using Centrifree® ultrafiltration devices to remove protein. This was used to generate the calibration curve to measure the protein unbound danofloxacin concentrations and for assay validation. Six replicates of 0.3, 3 and 7 μg/mL of danofloxacin in filtered EGM-2 were performed, and precision and accuracy were found to be 6.23–9.46 % and 90.5–97.9 %, respectively (Table 2). In order to assess drug binding to the collagen used to line the chip, collagen I (50 μg/mL) in physiological saline (Merck) was spiked at 0.15 and 1.5 μg/mL danofloxacin (*n* = 6 replicates) and followed the same steps as described above for the EGM-2. Precision and accuracy for the collagen matrix assay were found to be 2.2–3.5 % and 90.5–98.4 %, respectively (data not presented).

**Table 2:** Intraday precision and accuracy (*n*=6 replicates within 1 day) for filtered EGM-2 samples. Six filtered samples were spiked with danofloxacin at concentrations of 0.3, 3 and 7 μg/mL to validate the analytical assay for standard curve (0.05–10 μg/mL)

Analyte	Spiking concentration	Ave estimate concentration	SD	CV	Recovery
	(μg/mL)			(%)	(%)
		(μg/mL)			
danofloxacin	0.3	0.28	0.026	9.46	92.6
	3.0	2.94	0.183	6.23	97.9
	7.0	6.33	0.436	6.88	90.5

*SD*, standard deviation; *CV*, coefficient of variation

### Statistics

Statistical analysis was performed using GraphPad Prism v9.0.0 for Windows (GraphPad Software Inc., San Diego, CA). Replicates are detailed in figure legends. Data are presented as means ± standard deviation unless otherwise stated, where *n* represents individual chips. Significance is denoted as * *p* ≤ 0.05, ** *p* ≤ 0.01, *** *p* ≤ 0.001 and **** *p* ≤ 0.0001. Permeability and gene expression over time were analysed using ANOVA and Tukey’s multiple comparisons test for variability, once the normality of data had been confirmed. Protein binding of danofloxacin was analysed using a paired *t*-test.

## Results

### Assembly and culture of bLOC

Cultures were assessed in two ways up to 21 days at ALI following seeding onto the Emulate chip, visually for morphology under an upright light microscope and using IF to ascertain the presence of key differentiation or cell-specific markers. Light microscopy images of BPAEC and BBEC monolayers were taken from the outlet ports as depicted in Fig. 1A–B respectively (to enable clearer imaging of monolayers). BPAECs were confluent 24 h post seeding (Fig. 1A), whilst the BBECs also formed tight monolayers (Fig. 1B), with cilia observed in the epithelial channel from day 7 onwards (Fig. 1C iii, arrows). By day 14 at ALI, cells in the endothelial and epithelial channel were becoming over-confluent (Fig. 1C iv–v), with both channels showing extensive overgrowth at day 21 (Fig. 1C vi–vii), with complete coverage of the endothelial channel by BPAEC (depicted as a schematic in Fig. 1D).

### Mucin secretion as an indicator of BBEC differentiation under ALI

**Fig. 3:**
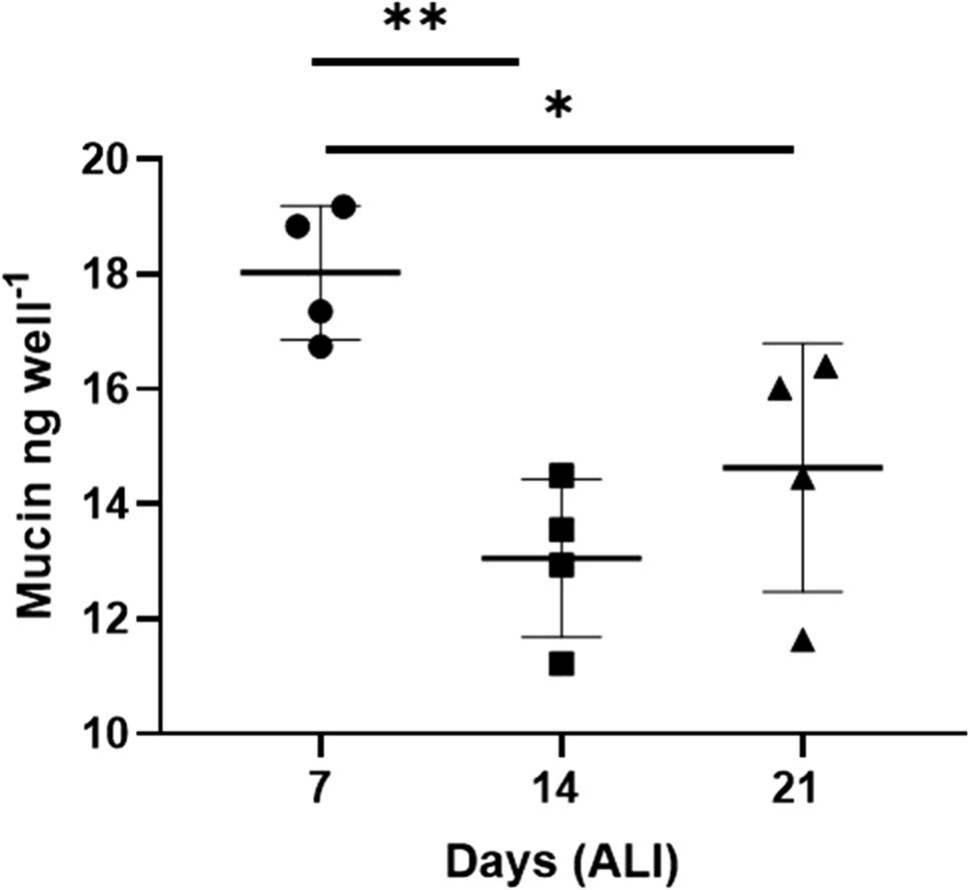
Mucin secretion was quantified using the lectin binding assay. Slight variation was observed between chips, with a significant difference observed in the means of all replicates between days 7–14 and 7–21 (ANOVA; **P = 0.005 and *0.039 respectively). Data presented as means ± SD; *n*=4

Mucus was already visible under the light microscope by day 7 of ALI, necessitating a twice weekly wash of each chip, in order to visualise the epithelial channel. Mucin secretion by goblet cells, as quantified in a lectin binding assay, was used as an indicator of basal cell differentiation and consequently mucus production of BBECs in the bLOC, sampling at days 7, 14 and 21 of ALI culture. By day 7, mucin detection as quantified by binding of the lectin isolated from *Triticum vulgaris* was already considerable. This remained so throughout the culture period up until 21 days, the last analysis point. Mucin quantity ranged from 13.6 ± 16.4 ng/mL, with the average quantity (of all chips) expressed at 7 days being significantly different to days 14 and 21 (*P*= 0.005 and 0.039, respectively; *n*=4) (Fig. 3).

### Immunofluorescence microscopy to ascertain layer integrity and protein expression of cell markers

**Fig. 4:**
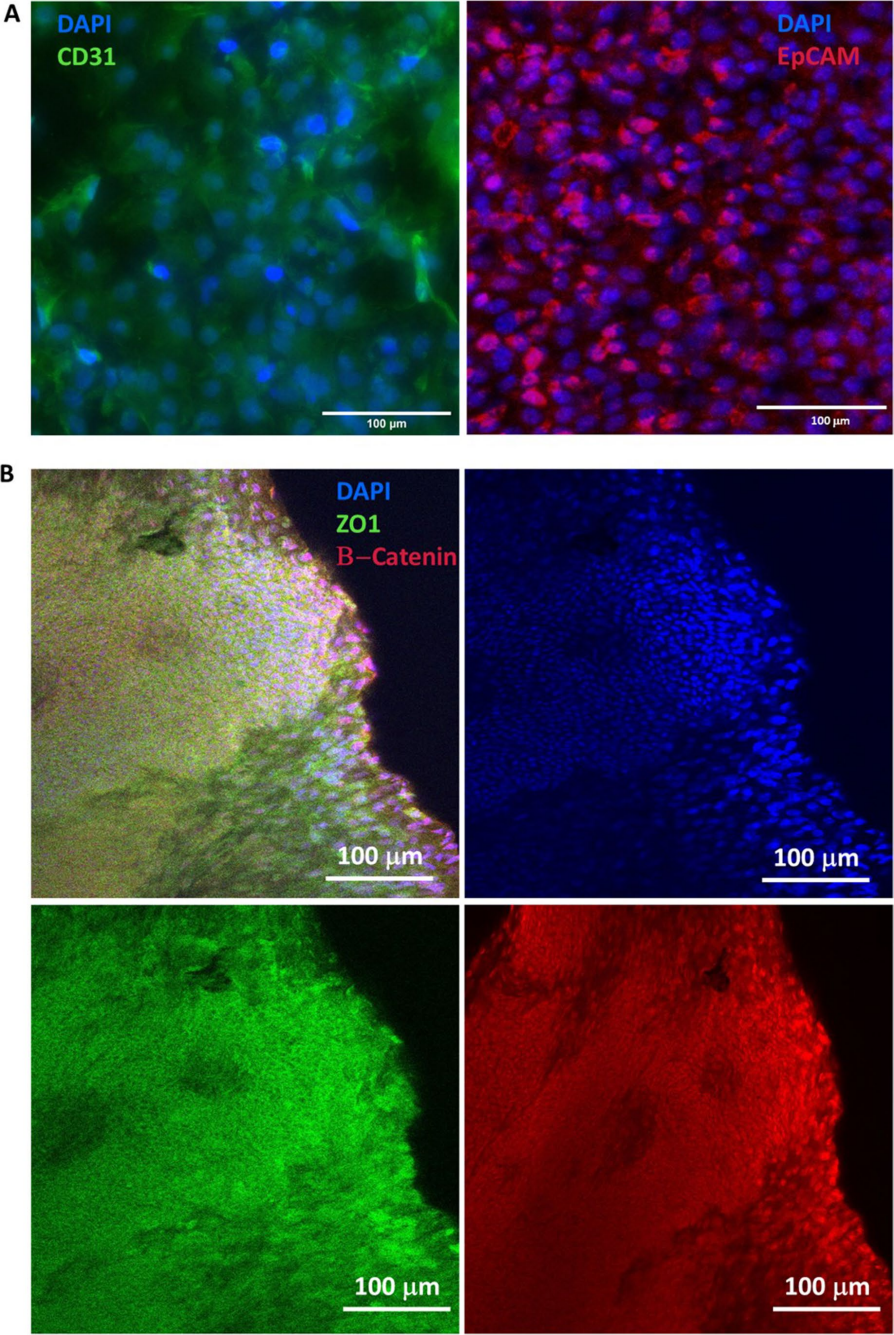
Immunofluorescence analysis of cell-specific and integrity markers. Both markers of endothelial cells (CD31) and epithelial cells (EpCAM) were observed in the endothelial and epithelial channels respectively (images acquired from the centre of the chip) (**A**). Cell layer integrity was demonstrated in the epithelial channel by the presence of the tight junction protein ZO-1 and adherens junction protein β-catenin (images acquired from the outlet port of the chip) (**B**). Scale bars as indicated on each tile

Immunofluorescence (IF) was used to demonstrate the presence of cell-specific markers EpCAM (epithelial cells) and CD31 (endothelial cells) (Fig. 4A) in the epithelial and endothelial channels respectively. The tight junction protein ZO-1 and adherens junction protein β-catenin were both demonstrated in the epithelial channel, indicating an integral layer of epithelial cells (Fig. 4B). Nuclear staining of β-catenin was observed on the leading edge of the expanding monolayer (image acquired in the outlet port of the chip), consistent with the protein’s role in the canonical Wnt signalling pathway.

### Gene expression of cell-specific markers over 21 days ALI

**Fig. 5:**
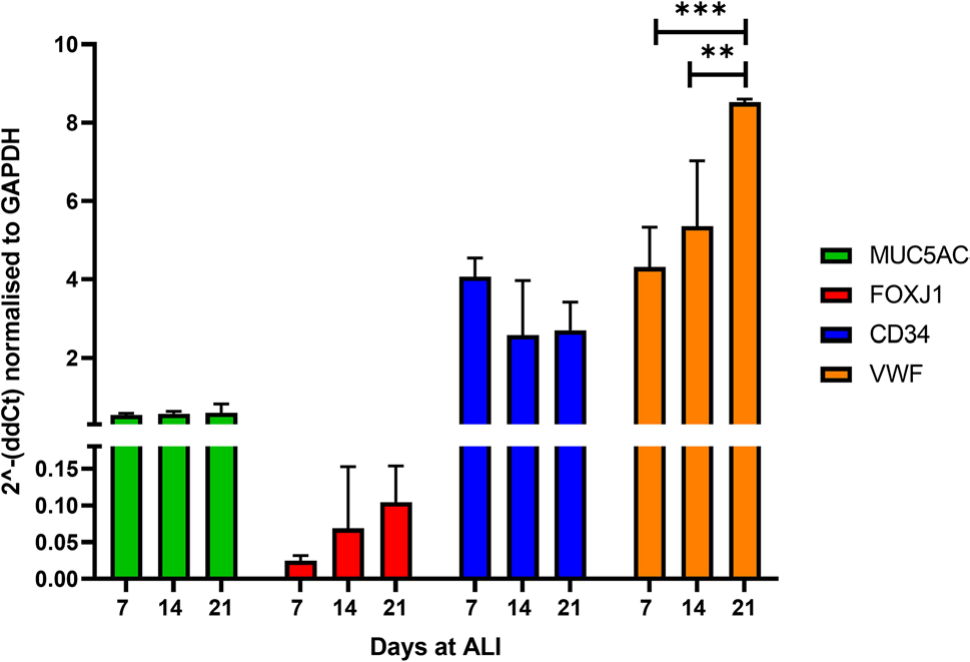
Expression of cell-specific markers at days 7, 14 and 21 days at ALI. Targets studied were *MUC5AC* (secretory cells), *FOXJ1* (ciliated cells), *CD34* and *VWF* (both endothelial cell markers). Expression of *MUC5AC* mRNA was consistent across days 7–21, as was the endothelial marker *CD34*. Expression of both the ciliated marker *FOXJ1* and endothelial marker *VWF* increased over the three time points studied, although *FOXJ1* was not found to be significant (ANOVA, ** *P* = 0.0029 and *** *P* = 0.0003). Data presented as means ± SD, *n*=2

In order to demonstrate physiological relevance, it is important to provide evidence of a heterogeneous population of seeded airway epithelial cells in vitro [21], arising from the differentiation of basal cells into ciliated cells and goblet cells [22–24]. This was accomplished in the current study by RT-PCR, targeting the ciliated cell marker *FOXJ1* and goblet cell marker *MUC5AC*. Variability of the endothelial markers *VWF* and *CD34* was also studied at 7, 14 and 21 days. Expression of *MUC5AC* was already consistent from day 7, showing no variation between this time point and day 14 or day 21, reflecting the finding during light microscopy analysis that mucus production was considerable by 7 days (Fig. 5). The expression of *FOXJ1* was shown to increase up to day 21, consistent with the observation of cilia at these times. These increases were not found to be significant. The endothelial marker *CD34* was found to be consistent between each time point studied, whilst VWF was found to increase from 7 to 14 and again from 14 to 21 days (*P* = 0.0003 and *P* = 0.0029, respectively; *n* = 2).

### Apparent permeability as measure of model integrity

**Fig. 6:**
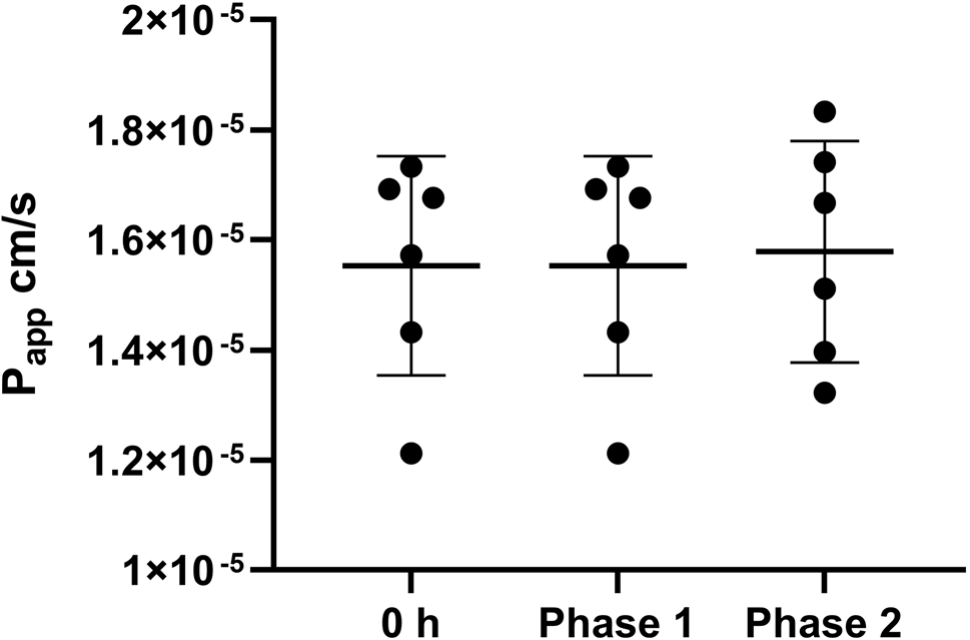
Apparent permeability (*P*_app_), calculated as a measure of integrity before, during and after danofloxacin treatment. No significant differences were detected before (0 h), during (phase 1) or after (phase 2) treatment (ANOVA, *P* = 0.968). Data presented as means ± SD, *n*=6

Integrity of the model was assessed by studying the paracellular transport of the permeability marker FITC-dextran (3–5 kDa), before, during (at the end of phase 1) and after the pharmacokinetics studies. The mean *P*_app_ values were determined to be 1.55 × 10^−5^ ± 2.0 × 10^−6^ cm/s at 0 h (pre danofloxacin treatment), 1.55 × 10^−5^ ± 2.0 × 10^−6^ cm/s at 24 h (phase 1 of danofloxacin pharmacokinetics study) and 1.58 × 10^−5^ ± 2.0 × 10^−6^ cm/s at 96 h (phase 2) (Fig. 6). No significant variability was found between time points (*P* = 0.968, *n* = 6), suggesting that the danofloxacin treatment and perfusion were not detrimental to the integrity of the model.

### UPLC-MS determined pharmacokinetics of danofloxacin in bLOC

**Fig. 7:**
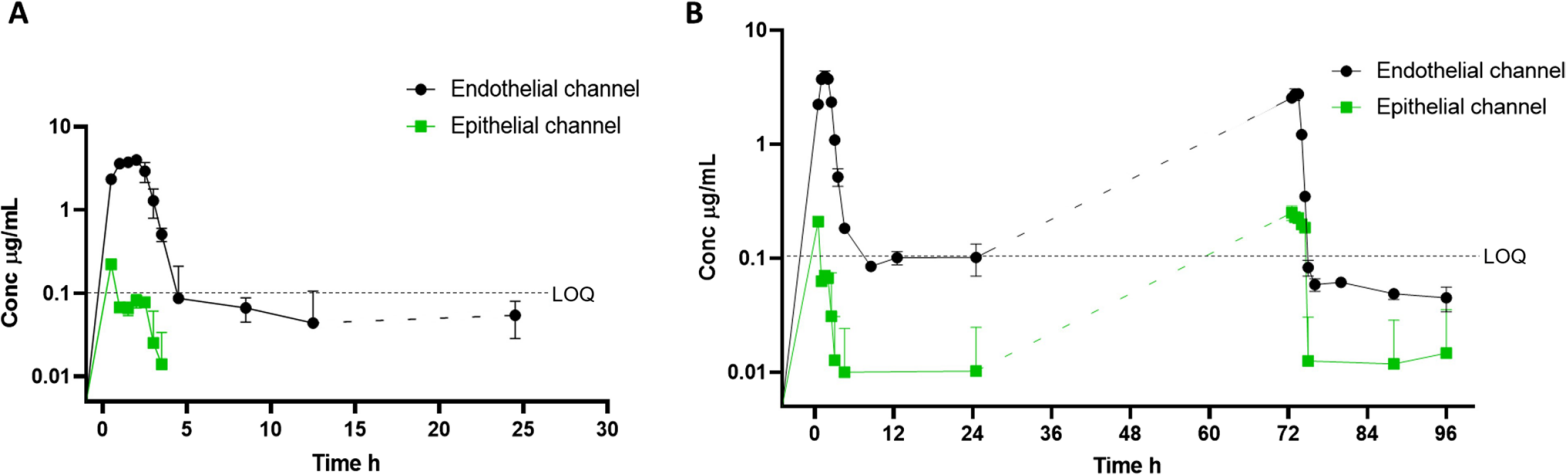
Study of danofloxacin endothelial to epithelial drug transport in a two-phase ‘pulse-chase experiment’. Danofloxacin was detectable in the endothelial channel until 3 h after its removal from the endothelial channel both in chips treated with one dose (phase 1, 0 h) (**A**) and chips treated with two doses (phase 1, 0 h and phase 2, 48–74 h) (**B**). Only epithelial channel samples acquired immediately post dosing contained detectable danofloxacin (LOQ = 0.1 μg/mL). Data presented as means ± SD, *n* = 4 (phase 1) and *n* = 2 (phase 2)

To assess the ability of the bLOC model to achieve a good IVIVC, a pulse-chase experiment was performed in two phases to follow endothelial to epithelial channel transport of the antibiotic danofloxacin (Fig. 2). The first phase consisted of a 2-h dose of danofloxacin (Fig. 7A) (*n*=4), with the second being a prolonged, 26-h dose (Fig. 7B) (*n*=2). These were designed to mimic the plasma concentrations observed during in vivo studies [25, 26], in different ages of calves. To allow comparisons between previously reported PK data (*C*_max_ and *T*_max_) obtained in the in vivo studies and the current in vitro study, the epithelial channel of the bLOC represented the pulmonary epithelial lining fluid (PELF), whilst the endothelial channel represented plasma (Table 3).

**Table 3:** Pharmacokinetic data acquired in previous in vivo studies [25, 26], directly compared with values obtained in the in vitro equivalent values obtained as part of the current study

Parameter	In vivo		In vitro (bLOC)	
	Plasma	PELF	Endothelial channel	Epithelial channel
*C*_max_ (μg/mL)	1.5 (6-month-old) [26]	1.61 (6-month-old) [26]	4.0	0.2
	1.79 (pre-ruminant) [25]	4.54 (3-week-old) [26]		
	2.12 (ruminant) [25]			
	2.2 (3-week-old) [26]			
*T*_max_ (h)	1.4 (6-month-old) [26]	2.0 (6-month-old) [26]	1.75	0.5
	1.44 (ruminant) [25]	2.4 (3-week-old) [26]		
	2.0 (3-week-old) [26]			
	3.13 (pre-ruminant) [25]			

Across the two in vivo studies previously reported [25, 26], the plasma *C*_max_ for danofloxacin ranged between 1.5 and 2.2 μg/mL, depending on the age of the calf. By way of comparison, the observed endothelial channel *C*_max_ was 3.9 ± 0.5 μg/mL for phase 1 (roughly double the concentration of danofloxacin introduced in the current in vitro study) and 2.9 ± 0.2 μg/mL for phase 2 doses (Fig. 7). Plasma *T*_max_ for the two in vivo studies ranged between 1.4 and 3.13 h, depending again on the age of the calf. In comparison, the median endothelial channel *T*_max_ was 1.75 h. Therefore, the current study achieved comparative values to the representative plasma PK in the endothelial channel of the bLOC.

In contrast, PELF *C*_max_ was only determined in one of the in vivo studies and ranged between 1.61 and 4.54 μg/mL, depending on the age of the calf. By contrast, the median epithelial channel *C*_max_ was 0.2 μg/mL. The PELF *T*_max_ for the in vivo study ranged between 2.0 and 2.4 h, depending again on the age of the calf. In comparison, the median epithelial channel *T*_max_ was 0.5 h (Fig. 7). In addition, danofloxacin was detected in the PELF of cattle up to 24 h after administration, whereas danofloxacin could only be detected in the epithelial channel samples for up to 2 h. Therefore, in contrast to the favourable plasma/endothelial channel IVIVC, we were unable to detect danofloxacin in most of the samples from the epithelial channel. Consequently, both the *C*_max_ and *T*_max_ were unrepresentative of the in vivo data. This resulted in an endothelial:epithelial *C*_max_ ratio of 20:1, which varied considerably from the in vivo plasma:PELF *C*_max_ ratio of between 1:1.1 and 1:2, depending on the age of the calf. Protein binding assays performed using spiked danofloxacin at 0.3, 3 and 7 μg/mL in the media (EGM-2) and in physiological saline (for collagen I) determined that bound danofloxacin concentrations were not found to be significant at any of the spike concentrations assayed, comparing with unbound (*P* = 0.27, paired *t*-test). Any protein binding of danofloxacin to proteins present in EGM-2 and to collagen I was thus determined to be non-consequential.

## Discussion

As the first bovine dynamic co-culture model of the proximal airway (search performed April 2022), it was crucial to demonstrate that freshly isolated BBECs were able to form integral tight junctions and form a heterogeneous population of ciliated and secretory (goblet) cells, hallmarks of a differentiated and polarised airway epithelium [27]. We focussed on morphometrics, barrier integrity, markers of differentiation and cell type and mRNA expression as a function of time. In the bLOC, the major cell-specific markers of epithelial (EpCAM) and endothelial (CD31) cells were both observed in 14-day-old cultures. The presence of a differentiated epithelium was demonstrated by expression of *MUC5AC* (secretory cells) and *FOXJ1* (ciliated cells). The increase of *FOXJ1* up to 21 days at ALI is indicative of the differentiation of basal cells to cells of a ciliated phenotype in its role as a progenitor during renewal of the airway epithelium [28–30]. Conversely, the expression of *MUC5AC* did not appear to alter over the 21 days. This reflected the excessive secretion of mucins as observed in the mucin secretion assay from an early time point (< 7 days at ALI), which necessitated multiple washes on the mucosal surface for subsequent analysis. The high quantities of mucin (*MUC5AC*) mRNA and protein expression were not surprising, given the sensitivity of secretory cells to shear stress, resulting in a cycle of excessive mucin production [31]. The increase in mRNA expression of the endothelial marker *VWF* (in contrast to the stable expression of CD34) was surprising, although this could reasonably be attributed to the response of endothelial cells to perfusion (again, shear stress) [32], or reflect the role of endothelial cell-secreted VWF in the repair of damage to the vascular endothelium [33].

The ability to accurately predict and evaluate key pharmacokinetic and pharmacodynamic parameters (PK/PD) of novel and existing therapeutics is arguably the holy grail for any investigator who subscribes to the ethos of the principles of the 3Rs (Replace, Refine, Reduce) [34]. Efforts to address the lack of non-human airway epithelial cell in vitro models required to conduct IVIVC studies do exist, including (but not exclusive to) those of Sreenivisan et al. (porcine) [35], McClenahan et al. (bovine) [36], and Cozens et al. (bovine) [5]. One common trait of these examples is their existence as monolayers, rather than co-culture models of two or more cell types, which would be closer to the in vivo situation they seek to model. Whilst McClenahan et al. were able to demonstrate detrimental effects of bacterial toxins on barrier integrity and cytokine release of endothelial cells in addition to epithelial cells, these cell types were studied in isolation. A number of more sophisticated ‘lung-on-chip (LOC) models’ have been developed to more closely recapitulate the in vivo physiology of the airway epithelium. These have notable advantages over the static arrangement of suspended ALI cultures or the organoid cultures pioneered by Clevers et al. [37, 38]. The application of stretch and perfusion to a co-culture system that enables multiple cell types to be grown in close proximity in different media enables researchers to more closely mimic in vivo interactions of cells with each other and introduced pathogens, therapies or treatments (reviewed by [15, 39]). The current study aimed to address the shortfall of species-appropriate (and dynamic) bovine in vitro co-culture LOC models by culturing primary bovine bronchial epithelial cells (BBECs) at an interface with bovine pulmonary arterial epithelial cells (BPAECs). The Emulate lung-on-chip was chosen on account of its flexible poly-dimethylsiloxane versus poly-ethylene terephthalate (PDMS) membrane. This chip has been previously shown to successfully mimic the effects of viral infection, inflammatory responses, and drug administration [40], whilst a relatively large pore size of 7 μm allows for the migration of immune cells in more sophisticated lung-on-chip model assemblies.

There were two objectives identified for this study. Assemble and characterise the model and test the model’s ability to recapitulate previous findings regarding the in vivo PK parameters of danofloxacin. A well-characterised synthetic fluoroquinolone antibiotic, danofloxacin is employed routinely in the treatment of *M. haemolytica* and *P. multocida* associated BRD, reported to achieve high concentrations in lung tissues with when administered intravenously or subcutaneously [16, 41]. To date, there have been no reports of in vitro models used to simulate endothelial to epithelial drug transport to the bovine lung, such as occurs in the administration of danofloxacin. Recent compartmental analysis was used to quantify post-dosage danofloxacin concentrations, including plasma and PELF [25, 26], for which the in vitro bLOC equivalents are represented by the endothelial and epithelial channels, respectively. The current study was not able to detect danofloxacin consistently in the epithelial channel under the conditions investigated. The data did, however, show that close to in vivo plasma PK was achieved in the endothelial channel in the form of *T*_max_ (Table 3). One possibility proposed for the inability to detect epithelial channel danofloxacin was the binding of the drug to plasma proteins, as observed previously [42]; however; a protein binding assay conducted using EGM-2 media found no significant difference in the concentrations of bound versus unbound. A further explanation for the failure to detect danofloxacin in the epithelial channel is the binding of danofloxacin to collagen I, used to coat the chip. Indeed, collagen I has been investigated as a drug delivery vehicle on account of its strong affinity for some drugs due to the presence of collagen binding domains [43]; however, protein binding studies with collagen I demonstrated minimal drug protein binding (4.8–18 % and 0.0–5.6 % binding for 0.15 μg/mL and 1.5 μg/mL, respectively). A third and most likely explanation is the dilution effect caused by the flow of media during sampling.

The current study placed emphasis on integrating perfusion and organ-on-chip technology into a model of the proximal bovine lung. Further studies should begin by the introduction of membrane stretch to simulate the cyclic mechanical strain imparted by breathing. This is particularly important for endothelial cells, where stress is now known to be critical for normal function and biochemistry [44, 45]. Additionally, previous studies have shown that the simulation of breathing through cyclic mechanical strain affects surfactant protein production in the alveolus and toxic and inflammatory responses of the lung to silica nanoparticles, in addition to enhancing epithelial and endothelial uptake of nano-particulates across multiple cell layers [46].

It is commonly accepted that IVIVC requires extensive mathematical manipulation [47], resulting in a formula that predicts in vivo values, rather than directly correlating with them. Since multiple physicochemical properties will affect solubility, stability and transport (a compound’s ionisation constant for example), the authors propose that these should be considered in further studies of the model. Even if the bLOC proves unsuitable for drug PK studies, it could still prove valuable for evaluating the interaction of pathogens with the bovine lung, particularly in light of the ability of microfluidic devices to impart mechanobiological forces upon the mucosal layer through dynamic stimuli such as fluid flow, stretch/strain and compression [39], as proven to induce significant phenotypical changes [48]. With this in mind, other researchers might build upon the PK data generated here by introducing BRD pathogens under cyclic mechanical strain, thus placing therapies such as danofloxacin into context and enabling intricate devolvement of the mechanism of action. The bLOC also lends itself to the repurposing of drugs for bovine or zoonotic respiratory disease, as demonstrated by use of human lung-on-chips in repurposing antimalarial therapies for the inhibition of COVID-19 infection [49]. Other researchers have dramatically changed the micro-landscape of the lung chip through the introduction of micro-curved porous membranes, generating a concave culture surface. Although most applicable to the alveolar region, it is worth exploring this concept for modelling the bronchiolar region of the lung, particularly since the use of a curved surface appears to affect cell density and epithelial layer thickness [50].

A noteworthy drawback of microfluidic systems such as LOCs (including the bLOC) is its complex design, incompatible with high throughput studies as required during drug discovery. The strength of any LOC lies in its ability to monitor parameters in real time, including but not limited to TEER measurements, secreted cytokines, and drug permeability. That said, the technology does exist to increase throughput of LOC systems [51], lending the technology to high throughput screening and studies of candidate compound toxicity and removing the LOC’s limitations to low throughput studies of physiological response and target validation. Indeed, several consortia now exist to aid application of this rapidly developing niche across the pharmaceutical industry and academia [52].

## Conclusions

We report here the development and characterisation of the first bovine lung-on-chip (bLOC). Although direct IVIVC for the model antibiotic danofloxacin was not possible in the current study, the characterisation of bLOC suggests that it could provide a viable in vitro organ model for drug and pathogen studies as an alternative to performing in vivo experiments in cattle.

## Data Availability

All data are available in their raw formats and without reservations upon request.
