# Publisher Correction: Development and evaluation of a bovine lung-on-chip (bLOC) to study bovine respiratory diseases

**DOI:** 10.1007/s44164-023-00041-4

**Published:** 2023-01-18

**Authors:** Diane F. Lee, Clare L. Thompson, Ronald E. Baynes, Hiroko Enomoto, Geof W. Smith, Mark A. Chambers

**Affiliations:** 1 School of Veterinary Medicine, University of Surrey, Guildford, UK; 2 Now at Sussex Drug Discovery Centre, University of Sussex, Falmer, UK; 3 Centre for Predictive In Vitro Models, School of Engineering and Materials Science, Queen Mary University of London, London, UK; 4 College of Veterinary Medicine, North Carolina State University, Raleigh, NC USA; 5 Zoetis Inc., Raleigh, NC USA

**Publisher Correction: In vitro models (2022) 1:333–346**

https://doi.org/10.1007/s44164-022-00030-z

Abstract

Purpose

Current air-liquid interface (ALI) models of bovine proximal airways have their limitations. They do not simulate blood fow necessary to mimic systemic drug administration, and repeated sampling requires multiple, independent cultures. A bovine lung-on-chip (bLOC) would overcome these limitations, providing a convenient and cost-efective model for pharmacokinetic or pathogenicity studies.

Methods

Bovine pulmonary arterial endothelial cells seeded into the endothelial channel of an Emulate Lung-Chip were interfaced with bovine bronchial epithelial cells in the epithelial channel. Cells were cultured at ALI for up to 21 days. Differentiation was assessed by mucin quantifcation, phase-contrast light microscopy and immunofuorescence of cell-specifc markers in fxed cultures. Barrier integrity was determined by FITC-labelled dextran 3–5 kDa permeability. To evaluate the model, endothelial-epithelial transport of the antibiotic drug, danofoxacin, was followed using liquid chromatography-mass spectrometry, with the aim of replicating data previously determined in vivo.

Results

bLOC cultures secreted quantifable mucins, whilst cilia formation was evident in the epithelial channel. Barrier integrity of the model was demonstrated by resistance to FITC-Dextran 3–5 kDa permeation. Bronchial epithelial and endothelial cell-specifc markers were observed. Close to plasma, representative PK data for danofoxacin was observed in the endothelial channel; however, danofoxacin in the epithelial channel was mostly below the limit of quantifcation.

Conclusion

A co-culture model of the bovine proximal airway was successfully generated, with potential to replace in vivo experimentation. With further optimisation and characterisation, the bLOC may be suitable to perform drug pharmacokinetic studies for bovine respiratory disease (BRD), and other applications.

This article is part of the Special Issue “Organ-on-a-Chip Technologies Network”, Guest Editor: Adrian Biddle, PhD, Queen Mary University of London, Barts and The London School of Medicine and Dentistry, UK. It was unintentionally published in issue 1/4–5 (2022).

You can access the article via this link: https://www.nature.com/articles/s44164-022–00030-z. We apologise for the inconvenience.

